# Effects of pairing health warning labels with energy-dense snack foods on food choice and attitudes: Online experimental study

**DOI:** 10.1016/j.appet.2020.105090

**Published:** 2021-05-01

**Authors:** Stephanie C.M. Asbridge, Emily Pechey, Theresa M. Marteau, Gareth J. Hollands

**Affiliations:** Behaviour and Health Research Unit, University of Cambridge, Forvie Site, Cambridge, CB2 0SR, UK

**Keywords:** Health warning labels, Image-and-text warning labels, Energy-dense snack foods, Explicit attitudes, Implicit attitudes, Conditioning

## Abstract

**Background:**

There is limited evidence concerning the potential effectiveness of health warning labels (HWLs) using images and text to depict possible negative health consequences of consumption, for reducing selection of energy-dense snack foods. Furthermore, the underlying mechanisms have received little attention; particularly effects on implicit attitudes, which previous work has shown may mediate the effect of aversive images on food choice.

**Aim:**

To assess the impact of pairing image- and text-based HWLs with energy-dense snack foods on a) the selection of, and b) implicit and explicit attitudes towards, those foods.

**Methods:**

Online experimental study with a representative UK sample (n = 1185), using a 2(Image/No Image) x 2(Text/No Text) factorial between-subjects design. Participants were randomised to one of four study arms, viewing snack food images paired with either: image-only HWLs, text-only HWLs, image-and-text HWLs, or no HWLs (control). HWLs concerned various negative health consequences of excess energy intake, such as heart disease and type 2 diabetes. The primary outcome was hypothetical food choice (energy-dense snack foods versus fruit), assessed post-intervention. Secondary outcomes were implicit and explicit attitudes.

**Results:**

Neither food choice nor explicit attitudes were changed significantly by any type of HWL. Implicit attitudes towards energy-dense snack foods were more negative after exposure to text-only or image-and-text HWLs. Both implicit and explicit attitudes predicted unique variance in food choice.

**Conclusions:**

This study suggests that short-term repeated exposure to HWLs paired with energy-dense snack foods may not consistently alter food choices, but can change implicit attitudes associated with food choices. Further laboratory and field studies are needed to more definitively assess the impact of HWLs on food selection and consumption in applied contexts and over time, as well as delineate underlying mechanisms.

## 1 Introduction

Excess intake of energy-dense snack foods is a key risk factor in the development of obesity and a range of non-communicable diseases, including type 2 diabetes, hypertension, coronary artery disease, cancers and stroke (Kopelman, 2007; Swinburn et al., 2004). As such, effective and scalable interventions to reduce intake of these products are urgently needed. One possible approach involves adding health warning labels (HWLs) to the packaging of health-harming foods (Clarke et al., 2020a). Such HWLs typically contain brief text and/or an image to highlight the potential health consequences associated with excess consumption of a given product. Evidence from tobacco research has consistently shown that HWLs have the potential to reduce consumption (Brewer et al., 2016). Both text-based and image-based HWLs have been used extensively as a public health intervention worldwide (World Health Organization, 2003), with growing evidence that incorporating graphic images may be more potent than text-only variants (Brewer et al., 2016; Noar et al., 2016).

Given evidence from tobacco that HWLs are an effective population-level intervention for reducing consumption, the possible application of HWLs to health-damaging food and alcohol products has been subject to an increased research focus, as well as continued interest among policymakers and other publics (Parry, 2014; Popova, 2016). While a recent systematic review (Clarke et al., 2020a) provides preliminary experimental evidence of the potential benefits of HWLs applied to food and alcohol products, the evidence base is small and incomplete. Most research to date has concerned sugar sweetened beverages (SSBs), finding that HWLs can reduce their selection and purchasing, with image-and-text HWLs most effective (Bollard et al., 2016; Donnelly et al., 2018; Mantzari et al., 2018).

However, compared with the literature on SSBs, there are few studies that assess potential behavioural impact of exposure to HWLs in the context of alcohol (Clarke et al., 2021b), and, pertinent to the current study, energy-dense snack foods (Rosenblatt, Bode et al., 2018; Rosenblatt, Summerrell et al., 2018; Clarke et al., 2020b). Importantly, the underlying mechanisms through which aversive warning imagery and text may have their effects have been little investigated in such domains, yet advancing mechanistic understanding is vital for harnessing and optimising potentially promising effects of interventions (Carey et al., 2019; Marteau et al., 2020). Whilst previous studies of HWLs applied to energy-dense snack foods have examined some possible mechanisms, including cognitive priming (Rosenblatt, Bode et al., 2018), reduction of automatic appetitive responses towards food cues (Rosenblatt, Summerell et al., 2018) and negative emotional arousal (Clarke et al., 2020b), none has been designed to examine the possible causal mediational role of attitudinal mechanisms, through which aversive imagery and text could impact upon food selection behaviour. This is merited because previous studies have demonstrated that changes in implicit attitudinal preferences - postulated to reflect automatic evaluations influenced by both cognitive and affective associations (Trendel & Werle, 2016) - can mediate effects on food choice of associating aversive images with energy-dense snack foods (Hollands & Marteau, 2016; Hollands et al., 2011). In general, experimental intervention studies examining mediation effects of implicit attitudes on food-related behaviours remain scarce (Sheeran et al., 2013).

Previous studies have used an experimental paradigm whereby image stimuli of food products are repeatedly paired with aversive image stimuli possessing negative affective valence, conceptualised as reflecting an evaluative conditioning process (Hofmann et al., 2010). Such controlled testing of the effects of repeated exposure to pairings of specific foods and aversive images can therefore enable understanding of how cognitive and behavioural effects may be realised more generally. However, because this previous work paired stand-alone aversive images with energy-dense snack foods, the generalisability of its results to responses to actual HWLs that include text statements is unknown. The current study therefore included realistic, plausible HWLs featuring text statements, in order to enhance the real-world applicability of the findings. These HWLs were comparable to those used in existing public health interventions, and were derived from an extensive development process (Pechey et al., 2020). The inclusion of text-only and image-only conditions enabled comparisons between the effects of different aspects of the HWL (the health message, the aversive image, and the combined effects of both) on behaviour and attitudes. As observed in the tobacco control literature, previous research focussing on energy-dense snack foods also suggests that HWLs containing graphic images in addition to text may be more effective in changing behaviour than text-only variants (Clarke et al., 2020a; Rosenblatt, Summerell, et al., 2018).

In addition, given there are conflicting findings in the literature about the extent to which implicit and explicit attitudes determine food choice (Karpinski & Hilton, 2001; Prestwich et al., 2011), the current study examined this to further inform understanding of whether implicit and explicit attitude change are similarly meaningful intervention targets. These attitudes may exert influence over behaviour in various ways (Perugini, 2005). First, there may be an additive pattern in which explicit and implicit attitudes predict unique variance in a given behaviour (Hollands & Marteau, 2016; Richetin et al., 2007). Second, in a double dissociation pattern, implicit attitudes are theorised to predict spontaneous behaviour and explicit attitudes to predict deliberative behaviour, but not vice versa (Conner et al., 2007; Perugini, 2005). Third, there may be an interactive effect, whereby implicit and explicit attitudes may interact synergistically to predict behaviour (Perugini, 2005). It may also be the case that one type of attitude mediates the effect of the other (Gawronski & Bodenhausen, 2011). In the current study, we hypothesised that both implicit and explicit attitudes would explain unique variance, in line with previous studies in a similar context (Hollands & Marteau, 2016). Finally, the study was conducted in a purposefully representative and relatively large general population sample, and both the study protocol and statistical analysis plan were pre-registered.

In sum, the present study investigated whether repeated exposure to HWLs highlighting negative consequences of excess consumption, associated with energy-dense snack foods, changed food-related choices and implicit and explicit attitudes. The specific, pre-specified study hypotheses were as follows:

1) Participants presented with HWLs will express a reduced preference for selecting energy-dense snack foods relative to fruit, compared to a control group not exposed to any HWLs. Given there is currently insufficient evidence in a food context to make a prediction about the relative potency of different types of HWLs, we hypothesised a similar effect of image-only, text-only and image-and-text HWLs.
2a) Participants presented with HWLs will express a relatively weaker implicit attitudinal preference for energy-dense snack foods, compared to a control group.
2b) Implicit attitudes will mediate any observed effect of the intervention on food choice.
3) Both explicit and implicit attitudes will significantly explain food choice behaviour.

## 2 Methods

### 2.1 Preregistration

The study protocol was preregistered on the Open Science Framework, prior to data collection, and a statistical analysis plan was preregistered before the data were inspected (https://osf.io/5gecb/).

### 2.2 Design

An experimental between-subject 2 × 2 factorial design, in which participants were randomised to one of four study arms, viewing snack food images paired with either: image-only HWLs, text-only HWLs, image-and-text HWLs, or no HWLs (control).

### 2.3 Setting

The study was conducted online using the Qualtrics survey platform.

### 2.4 Participants

Participants were recruited using a national research agency, Dynata (dynata.com), purposefully targeting a general UK population, with a representative range of age, sex and social grades. Inclusion criteria for participants were being at least 18 years old, able to read and write in English, having basic computer literacy, computer and internet access, and consuming energy-dense snack foods at least once a week.

A power calculation was conducted informed by but conservative in relation to a previous study (Hollands & Marteau, 2016) that found a small-to-moderate effect (*d* = 0.37) of a similar manipulation on snack food choice. Given power of 0.80 and an alpha level of 0.05, it was estimated that a total of 1096 participants (274 participants for each of four conditions) would be required in order to detect a small effect on the primary outcome (*F* = 0.10).

### 2.5 Materials

**Fig. 1:**
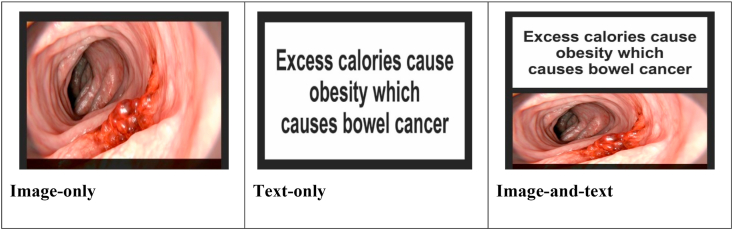
**Example HWL (here concerning bowel cancer) for image-only, text-only, and image-and-text conditions.***Image permissions from Shutterstock*.

The five image-and text HWLs used were those which elicited the greatest negative emotional arousal in a previous large-scale survey in a general population (described elsewhere: Pechey et al., 2020). These labels were then adapted for the image-only and text-only conditions of the current study (see Fig. 1). HWLs related to five possible negative health consequences of excess consumption of energy-dense snack foods, non-specified cancer, bowel cancer, heart disease, type 2 diabetes and obesity. A single image was used for each of the image-only and image-and-text HWLs, comprising a photographic representation of the human body's structure, anatomy or pathology (such as damaged organs or scenes of surgery). The final set of HWLs used are available at https://osf.io/5gecb/.

The images of fruit (apple, banana, grapes, orange) and snack foods (biscuits, cake, chocolate, crisps) used for the snack selection task, and those used for the conditioning procedure (biscuits, cake, chocolate, crisps, range of snacks) were sourced from an online database, Shutterstock. These images were highly similar in content to those used in a previous study (Hollands & Marteau, 2016) but of a higher image quality.

### 2.6 Measures

#### 2.6.1 Primary outcome measure

The primary outcome was a composite measure of hypothetical food choice, assessed post-intervention, combining 1) a voucher selection task plus 2) a snack selection task. In the voucher selection task, participants chose between one of two grocery vouchers (for Marks and Spencer, a UK grocery chain) ostensibly in reward for their time; one of which could be spent on fruit and the other on confectionery. In the snack selection task, participants chose one product from an array of eight food items, comprising four energy-dense snack foods (biscuits, cake, chocolate, crisps) and four fruits (apple, banana, grapes, orange) that they would most like to consume at that time. The images used in this selection task were the same images of snack foods used in the conditioning phase. Participants could also indicate that they did not want any of the presented options. As in previous studies, for each selection task, selection of a fruit option was scored ‘+1’, whilst selection of an energy-dense snack food option was scored ‘-1’ (with no preference as ‘0’). These scores were summed across the two selection tasks to produce the composite food choice score, with a value range consisting of −2 (two choices favouring energy-dense snack foods), −1, 0, +1, and +2 (two choices favouring fruit).

#### 2.6.2 Secondary outcome measures

*Implicit attitudes:* Implicit attitudes towards fruit and energy-dense snack foods were measured using a version of the Implicit Association Test (Greenwald et al., 1998) specifically adapted to assess relative preferences of fruit and energy-dense snack foods (Hollands & Marteau, 2016). The IAT requires participants to sort words relating to one of four categories using one of two response keys. The assumption behind the IAT is that participants will be faster at responding to categories which are strongly associated in memory when they share the same response key, relative to responding to categories not associated in memory which share the same response key. Comprising the four categories of the IAT were two target categories, each with five different examples: fruits (apple, banana, grapes, orange, fruits) and energy-dense snacks (biscuits, cake, chocolate, crisps, snacks) and two attributes, each also with five different examples. The two attributes were pleasant (happy, joy, rainbow, smile, and peace) and unpleasant (agony, death, pain, poison, and sickness). Each category and its associated examples appeared in text form only during the IAT, without any accompanying images. The IAT score (*D*), ranging from −2 to +2, was computed as a function of the difference in the mean response time between the two versions of the critical task (fruits-pleasant and snacks-unpleasant; fruits-unpleasant and snacks-pleasant), using scoring procedures described in Greenwald et al.’s (2003) revised algorithm for the IAT. This algorithm involves dividing each of the two computed differences by its associated pooled-trials standard deviation, and then averaging the two resulting quotients. The range of possible *D* scores is from −2 to +2, and it functions as an individual effect size assessment that is analogous to Cohen's *d*. Positive scores in this case indicating an implicit preference for fruit, whereas negative scores indicated an implicit preference for snack foods.

*Explicit attitudes:* Explicit attitudes towards fruit and energy-dense snack foods were assessed using five 7-point (1–7) semantic differential scales: ‘For me, eating fruit/snacks is … [not at all healthy-healthy], [good-not at all good], [bad-not at all bad], [unpleasant-not at all unpleasant], [enjoyable-not at all enjoyable]’, with reverse coding of these scores where appropriate. The total explicit attitudes score was produced by subtracting summed explicit attitude scores for snack foods from those for fruit (Perugini, 2005). As for implicit attitude scores: positive scores indicated a more positive explicit attitude towards fruit (and more negative explicit attitude towards snacks), whereas negative scores indicated a more positive attitude towards snacks (and more negative attitude towards fruit). For this measure, we conducted a reliability assessment applying the recommended approach to assessing reliability of difference scores that takes into account both reliability of its components and their correlations (Furr & Bacharach, 2008; Peter et al., 1993; Watkins (2020)). We did this for the current study and also compared it to data from our previous study (Hollands & Marteau, 2016) that used this same measure (noting that in both cases minimal correlations were observed between the fruit and snacks component scales of ≤ 0.06). For the current study, reliability for the difference score measure of explicit attitudes was found to be acceptable: reliability coefficient for the difference score measure *r* = 0.74 (explicit attitudes for fruit scale α = 0.88; explicit attitudes for snacks scale α = 0.62). For the 2016 study, the equivalent values were *r* = 0.79 (fruit scale α = 0.88; snacks scale α = 0.73).

Additional variables measured were age, sex, ethnicity, education, and self-reported height and weight (from which BMI was calculated). Each of the two fixed choice tasks (voucher selection and snack selection) were also examined separately as secondary outcomes, functioning as a sensitivity analysis.

### 2.7 Procedure

Ethical approval for the present study was granted by the 10.13039/501100000735University of Cambridge Psychology Research Ethics Committee (PRE.2018.105).

Participants completed initial screening questions and provided informed written consent. Inattentive participants were screened out via an embedded attention check (asking participants ‘When was the last time you travelled to Mars?’ [months ago/weeks ago/a few days ago/never], with any participant who responded anything other than ‘never’ being screened out) (Pechey et al., 2020). Thereafter, participants provided demographic information (age, sex, ethnicity, education level, height and weight). They were then randomised via the Inquisit software to view one of four conditioning slideshows on their computer (image-only HWLs, text-only HWLs, image-and-text HWLs, or no HWLs (control)).

The conditioning slideshow consisted of 110 trials (5 different snack food image trials, each shown 20 times in a random order; plus 10 trials containing only a target white circle stimulus, used as an attention check). Each trial lasted 2,500 ms in total, with each of the 100 snack food image trials comprising presentation of the snack food image for 1,000 ms, followed by an HWL or no image (in the control condition) for 1,000 ms. A blank screen (the inter-trial interval) was then presented for 500 ms. During this slideshow, participants were instructed to press the spacebar as quickly as possible whenever they saw a white circle stimulus. The white circle was interspersed at random among trials, functioning as an attention check to ensure participants remained at their computer for the duration of the slideshow. Any participants who did not press the spacebar at least once in response to the target stimulus were excluded from analysis.

Participants then completed the IAT, measuring implicit attitudes towards fruit and energy-dense snack foods. In the critical tasks of this test, stimuli from all four categories appeared in a random order and participants had to sort them into the appropriate category-attribute pair using the ‘E’ key for one pair (such as fruits-unpleasant) and the ‘I’ key for the other pair (such as snacks-pleasant). The category-attribute pairs were presented on opposite sides in the top corners of the screen and sorting errors were highlighted so that a correct response was required in order to move on with the task. The IAT was then followed by the measure of explicit attitudes towards fruit and snack foods. Finally, participants completed the behavioural measures of food choice; first, the snack selection task, followed by the voucher selection task. Finally, participants had the opportunity to leave comments in a free-text box. All participants received a full debriefing, informing them of the true purpose of the study – as upon entering the study they had been told only that it concerned thoughts and feelings about foods and the effect of viewing images on desirability of products - and that they would not receive the voucher that was previously offered, before being reimbursed £4 for their participation.

### 2.8 Data analysis

In line with previous work (Hollands & Marteau, 2016), data from participants meeting the following conditions were excluded: those who did not complete the study; those who attempted to enter the study more than once resulting in their assignment to multiple groups; and those who failed the stimulus response attention check during the intervention procedure because they did not produce any keyboard responses.

Following normality checks, the mean, standard deviation (SD) and 95% confidence intervals were calculated for each outcome and each HWL. A statistical analysis plan was pre-registered before the data were inspected (https://osf.io/5gecb/). 2 × 2 factorial Analyses of Variance (ANOVAs) were conducted to test for main effects of either Image, Text or an Image × Text interaction effect on the primary outcome (hypothetical food choice) and secondary outcomes (implicit and explicit attitudes). All statistical analyses were conducted using IBM SPSS v.25.

## 3 Results

Data from 1185 participants were included in the final analysis. 4425 participants initially clicked on the study link, 3138 of whom were either automatically screened out or dropped out before being randomised. The remaining 1287 participants consented to participate and were randomised to receive the intervention, of whom 1286 (99.9%) completed the study. Of the 1286 participants who completed the study, data from a further 101 participants were removed because they either attempted to enter the study more than once resulting in assignment to multiple groups and duplicate outcome data (33 participants), or failed the stimulus response attention check included in the intervention procedure because they did not produce any keyboard responses (68 participants).

Details of participant demographics can be found in Table 1, with the randomised groups well-balanced in their characteristics. Participant were 55% female, and ages ranged from 18 to 66 years. 54% had a university degree (slightly higher than for the UK population as a whole, but not markedly so, as for example, OECD 2014 data suggests 42% of UK citizens aged 25–65 have completed tertiary education). Details of outcome measures by group are in Table 2.

**Table 1:** Participant demographic characteristics.

Group	Image-only *(n* = *313)*	Text-only *(n* = *303)*	Image-and- text *(n* = *271)*	Control *(n* = *298)*	Total *(n* = *1185)*
Age, *M (SD)*	45.4 *(13.6)*	45.5 *(13.4)*	46.1 *(12.8)*	47.3 *(13.8)*	46.1 *(13.4)*
Sex- Female, *% (n)*	57.5 *(180)*	53.1 *(161)*	56.1 *(152)*	54.4 *(162)*	55.3 *(655)*
Ethnicity- White, *% (n)*	89.8 *(281)*	91.1 *(276)*	91.1 *(247)*	91.6 *(273)*	90.9 *(1077)*
Education - University Degree, *% (n)*	56.9 *(178)*	56.1 *(170)*	47.6 *(129)*	55.1 *(164)*	54.1 *(641)*
BMI, *M (SD)*	26.9 *(6.1)*	26.6 *(6.3)*	28.2 *(7.2)*	27.2 *(5.6)*	27.2 *(6.3)*

**Table 2:** Outcome measures.

	Image-only *(n* = *313)*	Text-only *(n* = *303)*	Image-and-text *(n* = *271)*	Control *(n* = *298)*	Total (*n* = *1185)*
Implicit Attitudes IAT Score (D), *M (SD)*^a^	.40 *(.56)*	.43 *(.59*59*)*	.47 *(.55)*	.32 *(.56)*	.40 *(.57)*
Explicit Attitudes, *M (SD*)^b^	9.26 *(6.54)*	9.42 *(6.52)*	9.21 *(6.70)*	9.21 *(5.88)*	9.28 *(6.41)*
Food Choice Measure (Choices of Fruit), *M (SD)*^c^	.12 *(1.62)*	.11 *(1.61)*	.08 *(1.68)*	.01 *(1.63)*	.08 *(1.63)*
Distribution of Values for Food Choice Measure, % *(n)*^c^					
−2	27.8 *(87)*	28.7 *(87)*	31.7 *(86)*	31.9 *(95)*	29.9 *(355)*
−1	8.9 *(28)*	7.6 *(23)*	6.3 *(17)*	6.0 *(18)*	7.3 *(86)*
0	19.8 *(62)*	20.8 *(63)*	18.1 *(49)*	21.8 *(65)*	20.2 *(239)*
+1	10.2 *(32)*	11.2 *(34)*	8.5 *(23)*	9.7 *(29)*	9.9 *(118)*
+2	33.2 *(104)*	31.7 *(96)*	35.4 *(96)*	30.5 *(91)*	32.7 *(387)*

a Range −2 to +2; More positive scores indicates a greater preference for fruit (versus snacks).

b Range −30 to +30: More positive scores indicates a greater preference for fruit (versus snacks).

c Range −2 to +2; More positive scores indicates a greater preference for fruit (versus snacks).

### 3.1 Primary outcome

A 2 × 2 factorial ANOVA was conducted to examine the effect of Text (Text/no Text) and Image (Image/no Image) factors on food choice. There were no significant main effects of either Text, *F*(1, 1181) = 0.097, *p* = .755, η_p_^2^ < 0.001, or Image, *F*(1, 1181) = 0.266, *p* = .606, η_p_^2^ < 0.001, and there was no significant interaction effect, *F*(1, 1181) = 0.427, *p* = .514, η_p_^2^ < 0.001.

### 3.2 Secondary outcomes

A 2 × 2 factorial ANOVA examining the effect of Text (Text/no Text) and Image (Image/no Image) on implicit attitudes yielded a main effect for Text, *F*(1, 1181) = 7.794, *p* = .005, η_p_^2^ = 0.007, such that implicit attitudes were more negative towards snack foods and more positive towards fruit when HWLs contained text (n = 574, *M* = 0.45, *SD* = 0.57) than when they did not contain text (n = 611, *M* = 0.36, *SD* = 0.56). Underlying this, there was a slightly larger effect observed for the image-and-text HWLs (M = 0.47, SD = 0.55) than the text-only HWLs (M = 0.43, SD = 0.59). There was no significant main effect of Image, *F*(1, 1181) = 3.435, *p* = .064, η_p_^2^ = 0.003, and no significant Text X Image interaction effect, *F*(1, 1181) = 0.227, *p* = .634, η_p_^2^ < 0.001.

The equivalent analysis for explicit attitudes found no significant main effects of either Text, *F*(1, 1181) = 0.058, *p* = .810, η_p_^2^ < 0.001 or Image, *F*(1, 1181) = 0.001, *p* = .979, η_p_^2^ < 0.001, nor a significant interaction effect, *F*(1, 1181) = 0.010, *p* = .919, η_p_^2^ < 0.001. Due to concerns about the scale structure of the explicit attitudes measures, we conducted an exploratory Principal Components Analysis, results of which are presented in Supplementary material. Examining the two fixed choice tasks (voucher selection and snack selection) separately as secondary outcomes gave the same results as seen for the composite primary outcome, with no observed effect of HWLs.

### 3.3 Relationships between explicit and implicit attitudes and food choice

A Pearson product-moment correlation analysis showed that implicit attitudes correlated with food choice, r = 0.19, p < .001, as did explicit attitudes, *r* = 0.404, *p* < .001. The two attitudinal measures also correlated with each other, *r* = 0.197, *p* < .001. A linear regression model was used to examine whether implicit and explicit attitudes towards energy-dense snack foods and fruit independently explained unique variance in food choice. An Implicit X Explicit attitudes interaction term was added as a second step. Given the correlation between the two attitudinal measures and the inclusion of the interaction term, all predictor attitude variables were centred in order to alleviate multicollinearity (Iacobucci et al., 2017). Results indicated that the model was a significant predictor of food choice (R^2^ = 0.177, *F*(3, 1181) = 84.49, *p* < .001), with both attitude types significantly explaining unique variance in food choice (implicit attitudes: standardised β = 0.12, *p* < .001; explicit attitudes: standardised β = 0.38, *p* < .001). The Implicit X Explicit interaction was not significant (standardised β = 0.02, *p* = .493).

## 4 Discussion

There was no evidence that either text-based HWLs or image-based HWLs affected food choice, meaning that Hypothesis 1 was not supported. There was partial support for Hypothesis 2a, as participants in the image-and-text and text-only conditions had more negative implicit attitudes towards energy-dense snack foods than those in the control condition, but image-only condition participants did not. As there were no meaningful intervention effects on food choice, it was not examined whether there was a mediation effect of implicit attitudes, so Hypothesis 2b could not be supported. Finally, implicit and explicit attitudes significantly explained unique variance in food choice, consistent with Hypothesis 3.

There are several possible explanations for why the HWLs were apparently not as potent for changing food choices as aversive images used previously within similar studies (Hollands et al., 2011; Hollands & Marteau, 2016). These will be examined in turn, and relate to first, the content and associated potency of the health warning stimuli, and second, the assessment of the primary outcome.

It is possible that HWLs – at least as configured here - are simply not sufficiently potent stimuli to change behaviour in a food product domain. Although there is not as yet sufficient evidence to demonstrate reliable and consistent impacts (for review see Clarke et al., 2020a), there is, however, emerging evidence of the efficacy of HWLs applied to real food and drink products in a range of contexts, including quasi-field (Grummon et al., 2019) and field (Donnelly et al., 2018) settings. An inherent lack of potency would also seem inconsistent with the effects on implicit cognitions observed in the current study, even though as in some other studies (e.g. Geng et al., 2013) this did not extend to any clear behavioural impact.

More specifically, the content of the warnings in the current study differed from those in previous studies using a similar paradigm (Hollands et al., 2011; Hollands & Marteau, 2016). In those studies, two of the five aversive images used featured images of people who would be classified as obese based on their BMI. Given these types of images of people with obesity are potentially stigmatising and therefore not appropriate for use in real HWLs (Johnstone & Grant, 2019), they were not used in the current study; an image of internal fatty tissue was instead used to represent obesity. However, such images may be more effective than other disease-related images and text statements in changing behaviour (Young et al., 2016), at least in the short-term.

The assessment of the primary outcome provides a further possible explanation for the somewhat discrepant results. In particular, while similar in concept and content, a new set of images of fruit and energy-dense snack foods were used for the snack selection task, in order to increase the previously poor image quality and visual consistency. It is possible that more directly comparable results would have been observed if an identical measure had been used. Furthermore, in order to ensure the snack selection task was identical between groups, the energy-dense snack foods therein did not have the different HWLs directly applied to them, contrary to typical studies of HWLs (Clarke et al., 2020a), where products or images of those products are themselves labelled. This logically reduces the salience of the association between HWLs and the energy-dense snack foods at the precise point of making a food choice, and so may reduce any effect of the manipulation. A final point pertaining to the primary outcome is that the controlled nature of the laboratory procedure used - being designed to elucidate possible mediational attitudinal mechanisms resulting from systematic exposure to stimulus associations - may inhibit the likelihood of observing behavioural effects, because these effects are assessed over the short-term within a single session. As such, any changes in behaviour (arising via, or unrelated to, implicit attitudes) that would be fully realised over the longer-term, or that rely on more intensive or tangible exposure to product-HWL associations, may not be captured. Given that multiple studies, including the current one, find that implicit attitudes as well as explicit attitudes predict and are associated with food choice behaviour (Prestwich et al., 2011; Richetin et al., 2007; Wang et al., 2015), it may be that the behavioural impact of changing implicit attitudes is only reliably observed with measurement over the longer-term.

While the observed effect of image-and-text and text-only HWLs on implicit attitudes is notable and furthers understanding of possible underlying mechanisms, the implications of such a statistically small effect on meaningful real-world change in cognitions or behaviour remain unknown and so should be interpreted with caution. Additional mechanistic studies in laboratory and field settings are needed to corroborate this finding. Studies designed to examine mechanisms but involving real food products, and with more intensive exposure over longer durations merit particular attention. Such studies would enable both better assessment of the potency of HWL interventions and of whether and how they could operate in part via changes in implicit cognitions. It is also recognised that a behaviour is likely to be driven to a greater degree by elicited implicit processes when characteristics of that behaviour or its context limit the likelihood of conscious reflection on the decision (Geng et al., 2013; Gibson, 2008; Hollands et al., 2016); with this not obviously the case for the current study. Uncertainties about the potential implicit mechanisms by which HWLs may have their effects reflect similar uncertainties in the tobacco literature, where conflicting findings have been reported regarding the impact of cigarette pack HWLs on implicit attitudes towards smoking (Macy et al., 2016; Van Dessel et al., 2018). It has been proposed that this may reflect the capacity of overly potent aversive images to evoke psychological reactance and undermine their intended effect (Erceg-Hurn & Steed, 2011; Ruiter & Kok, 2005; Van Dessel et al., 2018). As such, in developing and testing HWL interventions, it may therefore be important to establish an optimal level of image aversiveness.

We may have expected that the text used in the text-only and image-and-text HWLs would make the health consequences of consuming snack foods more explicit than in the image-only condition, in which this relationship is implied only through appearing sequentially within the slideshow. However, no effects of including text were seen for explicit attitudes so it is not clear that any such mechanism was elicited, and it was not directly assessed with other measures. Whilst it could help to explain the effect of text but not image components on implicit attitudes, explanatory text is not necessarily required, with previous studies having found effects of aversive images presented alone without text (Hollands & Marteau, 2016; Hollands et al., 2011). This possible inconsistency merits further attention.

The evidence for the effects of these kinds of aversive stimuli on explicit attitudes also appears inconsistent, with no intervention effects observed in the present study or in that of Hollands et al. (2011), despite significant effects on explicit attitudes reported by Hollands and Marteau (2016). It is possible that the order in which participants completed the attitudinal measures - the IAT first, followed by the measure of explicit attitudes - may have affected their responses (Phipps et al., 2019), so this may warrant further systematic investigation e.g. incorporating and evaluating additional counterbalancing procedures. However, both Hollands et al. (2011) and Hollands and Marteau (2016) also had participants complete the implicit attitudes measure before the measure of explicit attitudes, so this does not clearly account for differences in results.

Finally, in line with Hypothesis 3 and prior research (e.g. Hollands & Marteau, 2016; Phipps et al., 2019; Richetin et al., 2007), both implicit and explicit attitudes significantly explained unique variance in food choice. This suggests an additive pattern of attitudinal influence on behaviour relating to snack foods, with no significant implicit-explicit interaction effect being identified, indicating that the IAT measure of implicit attitudes has incremental validity and that there is discriminant validity between the measures of explicit and implicit attitudes. Practically, this suggests that interventions aiming to change such behaviours should target both explicit and implicit attitudes. More broadly, it further emphasises that advancing understanding of automatic or less conscious pathways to action should remain a key focus for intervention research (Marteau et al., 2012; Sheeran et al., 2013).

### 4.1 Strengths and limitations

Key strengths of this study are its relatively large, representative general population sample, and controlled experimental design, with preregistration of a detailed protocol and statistical analysis plan. To our knowledge, it represents the first attempt to investigate the effect upon implicit and explicit attitudinal mechanisms of systematic exposure to associations of food products with multiple different types of HWLs.

There are also some limitations. First, its online nature meant that the primary outcome measure was necessarily a simulated food selection, although it is plausible that fixed choice tasks in this context are more generalisable to online food shopping decisions, and, furthermore, the voucher choice component was presented ostensibly as having real-world implications.

Second, the unipolar nature of the IAT means that it assesses implicit attitudes towards energy-dense snack foods relative to fruit, so it was not possible to differentiate between individuals that liked both and those that disliked both, which could limit the predictive validity of the measure.

Third, the possibility of demand effects cannot be ruled out, given that the evaluative conditioning procedure was supraliminal and not subtle. However, participants were not aware of the conditions to which other participants were allocated, no effects of allocated group were observed on the behavioural primary outcome, and resistance to conscious self-presentation efforts is considered a strength of the IAT (Greenwald et al., 1998).

Fourth, prior research suggests that both images and imagery provoking text are more effective than non-imagery provoking text in changing implicit attitudes from positive to negative (Trendel et al., 2018). This could explain differential effects seen for different types of stimuli, and suggests that the current study could have benefited from systematic measurement and assessment of the nature and extent to which HWL text and images generate visual mental imagery.

Lastly, it is possible that the many repeated pairings of images of snack foods with HWLs within a short space of time during the slideshow may not adequately reflect real-world exposure to such HWLs, which we would expect to be less intensive. However, such research that investigates the effects of multiple repeated exposures to labels (albeit over a short time period) complements other research looking at less intensive and systematic exposures to HWLs in more naturalistic settings (Grummon et al., 2019; Clarke et al., 2021a) because it can systematically assess the degree of exposure that may be necessary to elicit any effect. Furthermore, many studies showing the effectiveness of tobacco HWLs on smoking behaviours have been conducted over longer time periods with multiple exposures (Brewer et al., 2016; Hammond et al., 2003). As such, while the short time period in which the exposures occurs in the current study may be a limitation of an online study, the many repeated pairings may approximate the overall level of exposure that we would expect to have an effect. It would therefore be important that further work on HWLs and snack foods is able to map the principles investigated in the present study onto more ecologically valid settings with more natural exposure to stimuli.

## 5 Conclusions

This study suggests that short-term repeated exposure to health warning labels (HWLs) paired with energy-dense snack foods may not consistently alter food choices, but provides important evidence that image-and-text and text-only HWLs can change implicit attitudes associated with food choices. Further laboratory and field studies are needed to more definitively assess the impact of HWLs on food selection and consumption in applied contexts and over time, as well as delineate underlying mechanisms.

## Ethics approval and consent to participate

Approved by the Psychology Research Ethics Committee of the University of Cambridge (Reference Number: PRE.2018.105). All participants provided informed consent.

## Consent for publication

Not applicable.

## Availability of data and materials

The dataset supporting the conclusions of this article is available on the Open Science Framework project page: https://osf.io/5gecb/

## Funding

Collaborative Award in Science from 10.13039/100010269Wellcome Trust (Behaviour Change by Design: 206853/Z/17/Z) awarded to Theresa Marteau, Paul Fletcher, Gareth Hollands and Marcus Munafò, and 10.13039/501100000272NIHR Senior Investigator Award (NF–SI-0513-10101) held by T. M. Marteau. The funders were not involved in the study design or data analysis. The views expressed in this publication are those of the author(s) and not necessarily those of the funders.

## Authors' contributions

SCMA, EP, TMM and GJH conceived the study and collaborated in designing the procedures. EP and SCMA coordinated the study and data collection. SCMA and GJH performed the data analyses. SCMA and GJH drafted the manuscript, with all authors providing critical revisions. All authors read and approved the final manuscript.

## Declaration of competing interest

The authors declare that they have no competing interests.
