# Supplementary material for: Effects of pairing health warning labels with energy-dense snack foods on food choice and attitudes: Online experimental study
Source: Appetite. 2021 May 1;160:105090. doi: 10.1016/j.appet.2020.105090 (PMC7895764; doi:10.1016/j.appet.2020.105090)
Supplement: Multimedia component 1 [file mmc1.docx]

**SUPPLEMENTARY MATERIAL**

**Exploratory Analyses of Explicit Attitudes Measures**

Due to concerns about the scale structure of the explicit attitudes measure, we conducted a Principal Component Analysis (PCA) on both the fruit and snacks scales with Kaiser normalization (including all components with Eigenvalues >1). The structure of the fruit scale was acceptable, with a single component extracted with loadings ranging from .75-.89. We also checked this in the data from our previous study that used the same measure (Hollands and Marteau, 2016), again finding a single component with loadings ranging from.78-.87). For the snacks scale, the 2016 data showed two components but with acceptable loadings for all items for the first component of .59-.74, suggesting it performed acceptably in this regard. For the current study data, however, two components were again extracted but loadings for the first component were sometimes weak and varied substantially from .27-.83. In combination, this suggests some uncertainty as to whether it is reasonable to combine all of the explicit attitude items for snacks.

Due to uncertainty about this measure, we therefore conducted further exploratory analyses, forming two separate explicit attitudes scales reflecting the results of the PCA. The first of these scales – Scale 1 - combined three items [not at all healthy- healthy], [good- not at all good], [bad- not at all bad], with acceptable reliability (Scale 1 for fruit α = .84; Scale 1 for snacks α = .73). The second scale – Scale 2 - combined the remaining two items [unpleasant- not at all unpleasant], [enjoyable- not at all enjoyable], again with acceptable reliability (Scale 2 for fruit α = .81; Scale 2 for snacks α = .77). Reliability coefficients for the difference score measures pertaining to these scales were also acceptable: r =.75 (Scale 1), r =.76 (Scale 2). We then conducted the original analysis of the effect of randomised group on explicit attitudes, and found similar results. For both Scale 1 and Scale 2, there were no significant main effects of either Text (Scale 1: F(1, 1181) = .497, p=.481, ηp2 < .001; Scale 2: F(1, 1181) =.373, p=.541, ηp2 < .001) or Image (Scale 1: F(1, 1181) =.153, p=.696, ηp2 < .001; Scale 2: F(1, 1181) =.324, p=.569., ηp2 < .001), nor a significant interaction effect (Scale 1: F(1, 1181) =.030, p=.862, ηp2 < .001; Scale 2: F(1, 1181)=.248, p=.619, ηp2 < .001).
